# Monitoring for COVID-19 by universal testing in a homeless shelter in Germany: a prospective feasibility cohort study

**DOI:** 10.1186/s12879-021-06945-4

**Published:** 2021-12-11

**Authors:** Andreas K. Lindner, Navina Sarma, Luise Marie Rust, Theresa Hellmund, Svetlana Krasovski-Nikiforovs, Mia Wintel, Sarah M. Klaes, Merle Hoerig, Sophia Monert, Rolf Schwarzer, Anke Edelmann, Gabriela Equihua Martinez, Frank P. Mockenhaupt, Tobias Kurth, Joachim Seybold

**Affiliations:** 1 Institute of Tropical Medicine and International Health, Charité - Universitätsmedizin Berlin, Berlin, Germany; 2 Department of Infectious Disease Epidemiology, Robert Koch Institute, Berlin, Germany; 3 Berliner Stadtmission, Berlin, Germany; 4 Labor Berlin-Charité Vivantes GmbH, Berlin, Germany; 5 Institute of Public Health, Charité - Universitätsmedizin Berlin, Berlin, Germany; 6 Medical Directorate, Charité - Universitätsmedizin Berlin, Berlin, Germany

**Keywords:** Homelessness, Homeless shelter, COVID-19, SARS-CoV-2, Pandemic, Infection control, Monitoring

## Abstract

**Background:**

Living conditions in homeless shelters facilitate the transmission of COVID-19. Social determinants and pre-existing health conditions place homeless people at increased risk of severe disease. Described outbreaks in homeless shelters resulted in high proportions of infected residents and staff members. In addition to other infection prevention strategies, regular shelter-wide (universal) testing for COVID-19 may be valuable, depending on the level of community transmission and when resources permit.

**Methods:**

This was a prospective feasibility cohort study to evaluate universal testing for COVID-19 at a homeless shelter with 106 beds in Berlin, Germany. Co-researchers were recruited from the shelter staff. A PCR analysis of saliva or self-collected nasal/oral swab was performed weekly over a period of 3 weeks in July 2020. Acceptability and implementation barriers were analyzed by process evaluation using mixed methods including evaluation sheets, focus group discussion and a structured questionnaire.

**Results:**

Ninety-three out of 124 (75%) residents were approached to participate in the study. Fifty-one out of the 93 residents (54.8%) gave written informed consent; thus 41.1% (51 out of 124) of all residents were included in the study. Among these, high retention rates (88.9–93.6%) of a weekly respiratory specimen were reached, but repeated collection attempts, as well as assistance were required. Around 48 person-hours were necessary for the sample collection including the preparation of materials. A self-collected nasal/oral swab was considered easier and more hygienic to collect than a saliva specimen. No resident was tested positive by RT-PCR. Language barriers were the main reason for non-participation. Flexibility of sample collection schedules, the use of video and audio materials, and concise written information were the main recommendations of the co-researchers for future implementation.

**Conclusions:**

Voluntary universal testing for COVID-19 is feasible in homeless shelters. Universal testing of high-risk facilities will require flexible approaches, considering the level of the community transmission, the available resources, and the local recommendations. Lack of human resources and laboratory capacity may be a major barrier for implementation of universal testing, requiring adapted approaches compared to standard individual testing. Assisted self-collection of specimens and barrier free communication may facilitate implementation in homeless shelters. Program planning must consider homeless people’s needs and life situation, and guarantee confidentiality and autonomy.

**Supplementary Information:**

The online version contains supplementary material available at 10.1186/s12879-021-06945-4.

## Background

People experiencing homelessness represent a vulnerable group with complex needs. Due to poor linkage-to-healthcare as well as lack of fulfilment of basic needs, they have higher occurrence of chronic mental and physical conditions, and higher mortality rates [1–3]. Exposure to severe acute respiratory syndrome coronavirus type 2 (SARS-CoV-2) might negatively affect their health, and further magnify these social and health inequalities [4]. Social determinants and pre-existing health conditions place homeless people at higher risk of severe coronavirus disease 2019 (COVID-19) [5, 6]. A study in Canada found that people with a recent history of homelessness required more frequent hospitalization and intensive care and had higher mortality rates compared with community-dwelling people [7]. The mobile nature of the community, high rates of substance abuse, informal sector employment or fear of involuntary hospitalization should be considered for screening, infection prevention, quarantining and treatment [4, 8, 9]. Access to health information, compliance with distance and hygiene rules, or self-isolation in case of symptoms can be a challenge for homeless people [10].

Congregate living settings in community shelters for homeless people that have shared bedrooms and sanitary facilities, facilitate the transmission of COVID-19 [11, 12]. The German infectious diseases notification system does not allow identification of homeless status and to our knowledge there is no data on the prevalence of COVID-19 among homeless people in Germany. A systematic review and meta-analysis on SARS-CoV-2 prevalence, transmission, health-related outcomes and control strategies in homeless shelters concluded that homeless people are at high risk of SARS-CoV-2 infection. During outbreaks in shelters, the pooled SARS-CoV-2 prevalence was 32% in homeless people and 15% in staff [12]. A low prevalence of symptoms at time of SARS-CoV-2 diagnosis was reported [13–15]. Even with low community prevalence, outbreaks of SARS-CoV-2 in homeless settings may lead to high attack rates [16]. Universal testing for COVID-19 at shelters is considered valuable when clusters occur. Moreover, preemptive testing for screening in shelters can be considered, especially when transmission is increasing in the general population [17].

COVID-19-related lockdown measures, contact restrictions and a decline of volunteer staff in homeless support services contributed to a general decrease of support of the homeless population globally [18, 19]. Provision of necessary measures for homeless people during the pandemic, e.g., shelter, basic needs and health care, was demanded by the United Nations Human Rights Commission and other organizations early on in the pandemic [20–24]. The German federal working group for homeless assistance explicitly called for the initiation of continuously open (24/7) shelters for homeless people [25]. To address these issues, the Senate of Berlin opened three temporary 24/7 shelters for people experiencing homelessness in May 2020, as well as a quarantine unit for SARS-CoV2 infected individuals [26].

Reverse transcription-polymerase chain reaction (RT-PCR) testing for SARS-CoV-2 is the current gold standard [27]. SARS-CoV-2 antigen rapid tests are relatively inexpensive and easy to perform at the point-of-care. They have a special value in congregate settings, where rapid test turnaround time is critical, even though they have lower sensitivity than RT-PCR [28]. Antigen rapid tests can detect the vast majority of SARS-CoV-2-infected persons within the first week of symptom onset and those with high viral load, making them a valuable tool to fight the transmission of SARS-CoV-2 [29]. Oropharyngeal or nasopharyngeal swabs for specimen collection are frequently perceived as uncomfortable and sometimes painful by the tested individuals. Compliance with repeated testing, applying oropharyngeal or nasopharyngeal swabs is likely to be difficult. Moreover, it requires numerous resources, like qualified staff and personal protective equipment. Evidence on the validity of less invasive sampling methods such as saliva collection or swabs taken from the nasal mid-turbinate or anterior nares, is increasing and was taken into account for example by the European Centre for Disease Prevention and Control, and the US Centers for Disease Control and Prevention [30, 31]. These methods can also be performed by individuals themselves.

The objectives of this study were (1) to assess the feasibility of regular, voluntary self-sampling for SARS-CoV-2 testing in a homeless shelter in order to monitor prevalence of infection and (2) to assess the feasibility of the approach to universal testing, especially in regard to the specimen collection and workload.

The results served as a basis for regular testing concepts, e.g., for the reopening of > 40 emergency overnight shelters with a capacity of > 1500 beds for homeless people in Berlin during the winter season 2020/21. Of these, almost 800 beds were located in 24/7 facilities that were established due to the pandemic [32].

## Methods

### Design, setting and participants

This was a prospective, feasibility cohort study with a mixed methods approach. Homeless people were recruited in one of three temporarily established 24/7 shelters in Berlin, Germany. The aim of the collaborative project between the Charité-Universitätsmedizin Berlin and the operator of the shelter, Berliner Stadtmission, was to enable shelter staff to conduct the study with a high degree of ownership and to integrate the study in their routine activities. The 24/7 shelter had 106 beds with shared bedrooms for up to 6 homeless adults. There was a fluctuation of shelter residents with about 10 new admissions per week. To be hosted, residents had to register on-site and fulfill requirements during their stay, such as mandatory temperature measuring and compliance with certain schedules. On the same location, there was an associated healthcare center with a newly established COVID-19 quarantine unit with 16 beds, for individuals not requiring hospitalization. The study was conducted over a period of 3 weeks between 9 July and 29 July 2020.

### Co-researcher team and recruitment

The study was initiated and supervised by a team of infectious diseases and public health professionals. The implementation was carried out by shelter and quarantine unit staff (co-researchers). The multilingual co-researcher team (German, English, French, Spanish, Russian, Polish, Romanian and Lithuanian) consisted of two coordinators, social work assistants, nurses, medical students, and physicians. All co-researchers were trained with a focus on good clinical practice (GCP) standards considering the vulnerability of the shelter residents. As part of the process evaluation, daily meetings during the first week enabled timely decision on readjustments of the monitoring design. The co-researchers also initiated an online communication platform to disseminate instructions and advice from the coordinators, to provide daily updates, clarify questions and share experiences.

Oral and written study information was provided to the residents to obtain written consent. Potential participants were informed in their native languages if available. Some residents were informed in groups (minimum of 2 persons), with the possibility of a personalized explanation afterwards. The consent form was available in German, English, French, Russian, Polish, Bulgarian, Romanian and Arabic.

The shelter residents were all ≥18 years old and were independently of symptoms eligible for the study. Residents were excluded from the study if they could not give adequate informed consent. Shelter staff in direct contact with the residents were also offered participation in this study, but their results were not included in the analysis.

### Specimen collection and analysis

The monitoring concept aimed to obtain a self-collected respiratory specimen of each resident on a weekly basis irrespective of symptoms. The interval of a weekly respiratory specimen was a pragmatic decision, as it was unclear which intervals would most efficiently prevent chains of infection. During the first week, saliva was used as a specimen. Residents were asked during the weekday’s morning round (6:30 am to 9 am) to spit into a tube through a straw. The procedure was guided by an instruction leaflet provided (Additional file 1), with additional staff support if needed. The targeted volume of saliva, marked on the tube, was 2 ml. The specimens were transported to the laboratory within 3–6 h of collection.

During the second and third week, a self-collected swab of tongue wiping, buccal mucosa and anterior nares was used, guided by an instruction leaflet (Additional file 2), with additional staff assistance if needed. The specimen was taken with the eSwab (Copan Diagnostics, Inc., USA) system with a nylon-flocked swab and liquid modified Amies medium. The study protocol permitted a collection regardless of the time of the day, as the specimen was in a tube with media and a short pre-analytical time was not of concern.

The participants were informed that in case of symptoms suspicious for COVID-19 the self-collected specimen did not replace a medical consultation at the ambulatory clinic and a swab taken by a health professional.

All samples were visually inspected to assess the proper closure of the tube, apparent abnormalities of the sample, and to perform sample volume estimation.

### RT-PCR analysis method

Especially saliva samples arrived in highly viscous condition in the laboratory. Pretreatment of these samples with DTT (Dithiothreitol) was carried out before RNA extraction on MagNA Pure 96 followed by real-time reverse transcriptase PCR (RT-PCR) on LightCycler 480 targeting the E-gene (LightMix® SarbecoV E-gene kit, Tib molbiol). eSwab samples were analyzed after addition of 1 ml Roche cobas PCR Medium on cobas 6800/8800 using the CE labeled cobas® SARS-CoV-2 assay (Roche) according to the manufacturer’s guidelines. An internal control in each sample as well as positive and negative controls were included in every run of both assays.

### Outcomes and measurements

Table 1 provides a summary of outcomes, measures/approaches, and methods of analysis corresponding to each objective. Regarding residents´ acceptability, the main measures were recruitment and retention rates. Recruitment rate was defined as the number of shelter residents that consented per number of residents who could be approached for participation. Retention rate was defined as the number of residents who were monitored with analysis of a respiratory specimens on a weekly basis compared with the number of recruited residents that were living in the shelter during that week.

Information on implementation barriers was collected with two evaluation forms that were developed together with the co-researchers. The forms were filled out by the co-researchers directly after the informed consent interview and specimen collection. The main variables recorded for the informed consent process were: language, duration of the interview, questions and doubts mentioned by the resident, difficulties as perceived by the co-researcher team, and suspected reasons for non-participation. The main variables recorded for the specimen collection were: number of attempts needed to collect the sample, difficulties reported by the resident, difficulties observed by the co-researcher team, and reason for non-collection of a specimen.

A final focus group with the co-researchers took place to discuss the feasibility of the overall approach. Based on experiences from the feasibility study, it was discussed to which extent the monitoring design needs to be refined for future implementation in similar settings. A quantitative structured questionnaire addressed recommendations for further implementation, team composition, sample collection, the essential content of the informed consent interview, assumed challenges for further implementation and critical appraisal of the pilot study. Moreover, protocols and notes of all meetings and trainings, the focus group discussion, and the online communication platform of the co-researcher team were considered for process evaluation and continuous adaption of the study design.

**Table 1:** Summary of outcomes, measures/approaches, and methods of analysis corresponding to each study objective

Objectives	Outcomes	Measures/approaches	Methods of analysis
Feasibility of study implementation	Residents' acceptability	- Recruitment rate - Retention rate - Evaluation forms - Focus group (staff)	- Descriptive analysis - Content analysis
	Implementation barriers and facilitators	- Evaluation forms - Focus group (staff) - Continuous feedback *via* online communication platform, calls, and field visits	- Descriptive statistics - Content analysis
	Staff acceptability	- Evaluation form - Focus group (staff)	- Descriptive statistics - Content analysis
Feasibility of study methods	Specimen acceptability	- Visual inspection of specimen - Evaluation forms - Focus group (staff)	- Descriptive statistics - Content analysis
	Workload	- Evaluation form - Focus group (staff)	- Descriptive statistics - Content analysis

### Data analysis

We used descriptive statistics to summarize recruitment, retention, and baseline characteristics, as well as to compare residents who consented and declined participation by age, sex, and language of the consultation. The data analysis of the process evaluation was based on the framework method [33]. It is a systematic approach that is applicable for qualitative health research in multi-disciplinary teams. Even if co-researchers were not familiar with qualitative research methods, they could be involved in the analysis process that was guided by experienced qualitative health researchers. After a first assessment of the qualitative data, categories and descriptive labels (codes) were identified and applied to the data (see Additional file 3). A combined approach (deductive and inductive) was used for anticipating unexpected aspects or events. Further information, according to the consolidated criteria for reporting qualitative research (COREQ), is given in Additional file 4.

### Ethics

This study was approved by the ethics committee of the CharitéUniversitätsmedizin (No.: EA4/141/20). The co-researcher team was sensitized to the dependencies between them and the shelter residents that might cause research participation coercion. Residents were explicitly informed that positive RT-PCR test results for SARS-CoV-2 would not allow a further stay at the 24/7 shelter, but that a 14-day isolation at the quarantine unit on site would be offered including food and television. Medical care, including substitution or alcohol as needed, would be provided by the medical staff of the ambulant clinic to which the quarantine unite was attached. Social workers would be available for any social or work-related aspects. Furthermore, participants were informed that a positive test result would be immediately reported to the responsible local health authorities according to the infectious disease act [34]. Notification includes personal details such as name and birth date of the person. Although the confidentiality of medical information could be assumed, this information was considered particularly relevant for persons without valid residence status.

## Results

### Recruitment and barriers

**Fig. 1:**
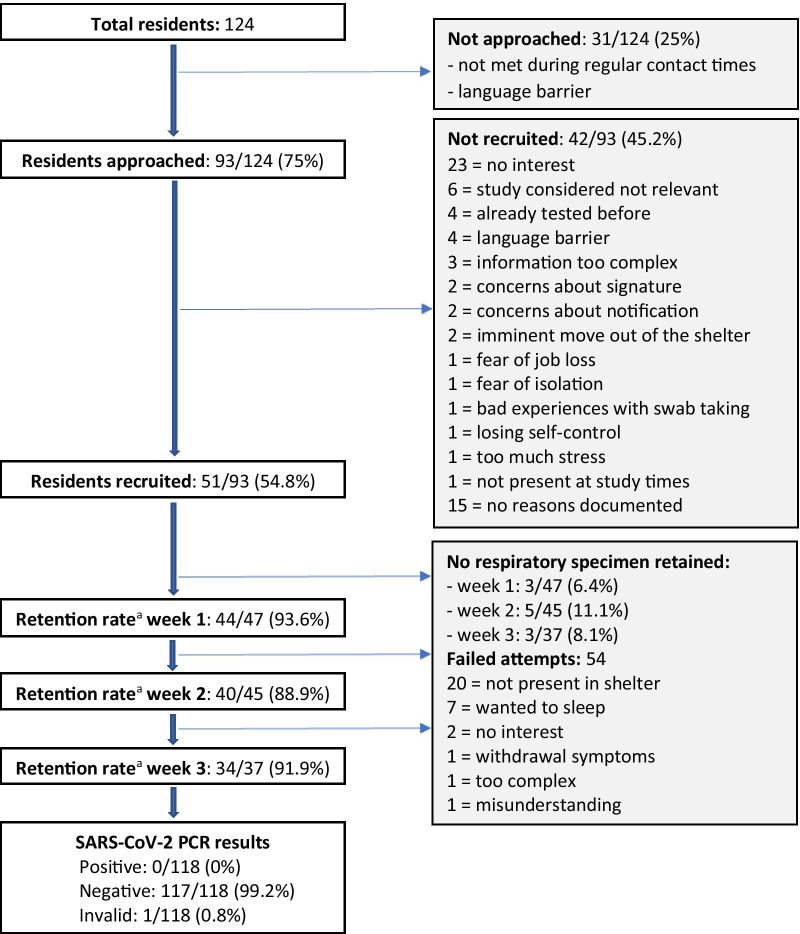
Study flow diagram with reasons for non-recruiting and non-retaining of residents for a weekly respiratory specimen. ^a^Retention rate: number of residents monitored with analysis of a respiratory specimens per week compared with the number of recruited residents that were still living in the shelter during that week

Due to fluctuation, 124 residents were living in the shelter with 106 beds during the three-week study period. Figure 1 shows the study flow with reasons of non-recruiting and non-retaining of shelter residents for a weekly respiratory specimen. Ninety-three out of 124 (75%) residents were approached to participate in the study. Thirty-one out of 124 (25%) could not be approached, either because they were not available during recruiting times, or because language mediation was needed and was unavailable. Demographic data of those was not available.

Baseline characteristics of the potential participants and the informed consent process are displayed in Table 2. Seventy-four individuals (79.6%) were male, with a male:female ratio of 4.9:1; median age was 47 years (IQR 34–54), with a range from 21 to 86 years. Information of participants took place mainly in the evening (94.6%). One third (33.3%) received the information in a group. Median estimated duration of the information was 10 min (IQR 5–10). 55.9% of the potential participants received information in other languages than German, of which all were available within the co-researcher team: Russian (18.3%), English (17.2%), Polish (12.9%), Romanian (6.5%) and French (1.1%).

Frequently asked questions by the residents addressed the general procedure (n = 3), and aim of the study (n = 1), communication of results (n = 2) and times of specimen collection (n = 2). Some had concerns about the use of personal data (n = 2) and signing the consent form (n = 1). Difficulties perceived by the co-researcher team were communication (n = 9), uncertainty of the resident (n = 3), move out of the shelter (n = 2), and lack of interest (n = 2). A written informed consent could be obtained from 51 out of 93 (54.8%) of the potential participants.

Consenting participants were comparable to non-consenting participants by age and spoken language. There were more women among the non-consenters (n = 9, [21.4%]) than among the consenters (n = 6, [11.8%]). The reasons for refusing participation, as perceived by the study team, are shown in Fig. 1.

**Table 2:** Baseline characteristics of residents approached for participation and of the informed consent process

		Residents approached (n = 93)
Sex	Female	15 (16.1%)
	Male	74 (79.6%)
Age, median, years (IQR)		47 (34–54)
Daytime of information	Morning	2 (2.2%)
	Noon	0
	Evening	88 (94.6%)
Individual information		59 (63.4%)
Group information		31 (33.3%)
Language mediation	Yes	53 (57%)
	No	37 (39.8%)
Language of consultation	German	37 (39.8%)
	Russian	17 (18.3%)
	English	16 (17.2%)
	Polish	12 (12.9%)
	Romanian	6 (6.5%)
	French	1 (1.1%)
Duration, median, minutes (IQR)		10 (5–10)
Questions of participants	3 = general procedure of the study 2 = communication of results 2 = times of specimen collection 1 = potential costs 1 = aim of study	
Concerns of participants	2 = use of personal data 1 = giving signature	
Difficulties perceived by co-researcher team	9 = difficulties in communication (language barriers) 3 = uncertainty 2 = moving out soon 2 = lack of interest	
Consent to participation	Yes	51 (54.8%)
	No	42 (45.2%); 5 later withdrawn

Data are n (%); age and duration with median (interquartile range). Missing data: sex (n = 4), daytime (n = 3), individual/group information (n = 3), language spoken (n = 4)

### Retention and acceptability

During the first week, a respiratory specimen (saliva) from 44 out of 47 (93.6%) residents who had consented and were living in the shelter during that week could be retained for SARS-CoV-2 testing by RT-PCR. During the second and third week, a respiratory specimen (self-collected swab of the tongue, buccal mucosa, and anterior nares) from 40 out of 45 (88.9%) and from 34 out of 37 (91.9%) residents, respectively, could be retained for testing.

In many cases, repeated attempts to collect the specimen were necessary. The co-researcher team reported fifty-four attempts of specimen collection where the test could not be performed, mainly because the resident was not present in the shelter (n = 20) or because the collecting time of the samples in the morning was inconvenient (n  =  7). Furthermore, in some cases, a specimen collection was not possible due to a lack of interest (n = 2), withdrawal symptoms (n = 1), misunderstanding of study procedure (n = 1) or the complexity of sampling (n = 1) despite assistance (Fig. 1). Dry mouth and, therefore, long duration of sampling was mentioned to be difficult in 5 cases as well as the use of a straw for saliva collection (n = 2). According to the focus group, difficulties during the specimen collection were underreported in the evaluation forms. Early morning collection of samples was perceived as a burden for both residents and staff. A flexible collection of samples during the day would have been preferred.

For the self-collected swab of the tongue, buccal mucosa and anterior nares, 24 out of 52 (46.2%) residents refused to place the swab in the nares after having been in the oral cavity for hygienic reasons or perceived as uncomfortable. The co-researcher team pointed out that lack of fine motoric skills made the self-collection difficult in some cases. All reasons reflect the perspective of the co-researcher team, not of the residents.

### Specimens and results of RT-PCR analysis

Sample self-collection was guided by an instruction leaflet provided to the participants (see Additional files 1, 2). The targeted volume of saliva was 2 ml per specimen and marked on the collection tube. The median volume collected was 1.7 ml (IQR 0.9-2), thereof 7 specimens with a volume of less than 0.5 ml. The visual inspection of the specimen revealed the following abnormalities: empty tube (n = 1), tube not adequately closed (n = 1), obvious external contamination with saliva (n = 1), specimen consistent and viscous (n = 6). The visual inspection of the self-collected swab from tongue, buccal mucosa, and anterior nares revealed the following abnormalities: tube not adequately closed (n = 1) and media in tube incomplete (n = 1). The highly viscous nature of saliva samples (at least at the time when samples arrived in the laboratory) made it necessary to pre-treat samples before routine molecular diagnostic testing. Thus, this sample type is more prone to invalid test results than the eSwabs used in the second and third week. This is especially true for sample processing in fully automized high throughput testing systems like Roche’s cobas 6800/8800.

During the study period, no resident was tested positive for SARS-CoV-2 by RT-PCR. 117/118 (99.2%) specimens were tested negative. One analysis was considered invalid as the sample tube appeared to be empty.

### Workload

During the 3-weeks of the pilot study, a total of about 274 person-hours were invested by the co-researcher team for training and meetings (39.1%), obtaining informed consent (32.8%), weekly sample collection including preparation of material (17.5%), project coordination (7.8%) and data maintenance (2.8%). The workload was distributed between 2 project coordinators and 20 team members. The working hours of the supervision team were not included in the analysis. In the focus group and final evaluation questionnaire it was emphasized that the workload was high and difficult to manage together with other routine activities.

### Feasibility and critical appraisal

Seventeen out of 22 co-researcher team members participated in a final focus group discussion. Overall, the implementation of the monitoring was perceived to be valuable and a good experience, but work-intensive. Only 1 out of 17 team members stated that monitoring would not be possible during the winter season in the emergency night shelters. In the critical review of the results, the team reported that barriers for recruitment and retention were underestimated due to incomplete documentation in the evaluation forms. Non-availability of a common language, particularly Bulgarian, Lithuanian, Czech, and Vietnamese, was the main barrier to participation. Provision of written negative test results could have increased participation among the residents. Also, the presence of people in the team who were familiar to the residents was crucial and increased trust and willingness to participate.

Twelve team members participated in the final evaluation with a structured questionnaire. Eleven out of 12 preferred the self-collected swab for the following reasons: more flexible times of collection (n = 6), more hygienic (n = 5), less complicated (n = 5), and faster to collect (n = 4). Visual instruction leaflets for sampling [see Additional files 1, 2] were perceived as suitable by 7 team members, whereas on-site demonstration and direct assistance during the collection was perceived to be helpful or necessary by 10 team members. The use of video or audio formats was suggested (n = 6) as optimization of information and instruction for sample collection.

Table 3 provides selected themes with illustrative quotes that were emphasized by the co-researchers in the focus group, online platform, or final evaluation questionnaire. In terms of acceptability by the residents, allowing flexible times of sample collection or of providing information, as well as adapting the written information in a more precise, adapted language was suggested.

**Table 3:** Selected quotes emphasized by the co-researchers in the focus group, online platform, or final questionnaire

Theme	Quotes
Interaction with residents	The project team should include people who have already built up trust to the homeless community
	Be prepared for multiple languages and guarantee barrier-free communication
	Use audio- and video formats for provision of information
	Informed consent and sample collection may be influenced by intoxication of residents
	It's a nice way to talk to people you didn't know before. Many were happy about the conversation
Willingness for participation	During the cold season, homeless people might have other priorities, consider needs and daily routine of the residents in the planning
Specimen collection	Patience and understanding for repeated instructions are needed
	With more flexible times of sample collection, we could have reached more residents
	Without assistance, the collection would have not been possible for several residents
Staff and workload	Good and continuous communication among the health workers, social workers and language mediators, as well as supervision was essential
	Additional staff is needed due to high workload of monitoring activities
	Benefits for residents and staff should be identified and emphasised. The monitoring gave me a feeling of security in the shelter
Ethical considerations	Information on data use and consequences of a positive test result should be transparent
	Accept a “no”, take people as they are

## Discussion

### Summary of main results

Fifty-one out of 124 (41.1%) residents were recruited for this study. Among these, high retention rates (88.9–93.6%) of a weekly respiratory specimen could be reached during the 3 weeks, however, in many cases repeated attempts to collect the specimen were necessary. A self-collected swab of the tongue, buccal mucosa and anterior nares was considered easier and more hygienic to collect than a saliva specimen. Several of the saliva samples showed a reduced volume and high viscosity making them less suitable for standardized molecular diagnostic testing. On-site demonstration and assistance were frequently necessary with both saliva and swab in order to obtain an adequate specimen. A considerable number of residents (n = 25, [46.2%]) refused to place the swab in the nares after having placed it in the oral cavity due to hygienic reasons or because it was perceived as uncomfortable. All specimens tested were negative for SARS-CoV-2 by RT-PCR during a period of low community transmission in Berlin in July 2020 [35].

There were more women among the non-consenters (n = 9, [21.4%]) than among the consenters (n = 6, [11.8%]). Two-thirds of conversations were conducted with language mediation, mainly in Eastern European languages. Language barriers were reported to be one of the main reasons for non-recruitment and difficulties in the recruitment process. Barriers for participation were lack of interest to participate, perception that study information was too complex, concerns about the use of personal data, and providing a signature. Flexibility of sample collection schedules, the use of video and audio materials, and concise written information were the main recommendations of the co-researchers for future implementation. Sufficient human resources were considered essential for the successful implementation of a monitoring concept, which also allows the individual needs of the residents to be considered.

### Strengths and limitations

An understanding of the challenges and issues related to recruitment and retention—especially in a so called hard-to-reach population—is important and can help policy makers to foresee strategies to overcome these issues. In Berlin, this was relevant for more than 40 emergency overnight shelters during the winter season [32].

The residents of the 24/7 shelter were not representative of all people experiencing homelessness in Berlin. People in highly precarious situations (e.g., with psychiatric disorders or irregular residence status) were unlikely fully represented, due to the fact that individuals had to register at the shelter with their name and fulfill certain requirements during their stay.

Only 41.1% of all residents were included in the study. Some of the residents could not be found during testing times or could not be addressed due to language barriers. The need of a signature in the study consent form may have discouraged some individuals from participating. Furthermore, at the early stage of the pandemic residents were probably not well informed about COVID-19. More time to inform residents before the start of the study would have been beneficial. The high retention rates among study participants may not be fully generalizable, as the co-researchers put huge efforts in conducting the study, demonstrated by the repeated attempts to collect the specimens. The analysis of recruitment and retention barriers may be biased, as it relied on observations made by the co-researchers. Lack of interest to participate or indifference to the topic might have different reasons such as lack of adequate information, or other basic priorities. People experiencing homelessness were not involved in the planning, implementation, or the evaluation of this study due to lack of time and resources. However, it would have contributed to the quality of the study if the perspective of the study participants had been included.

### Implications for future SARS-CoV-2 testing concepts

COVID-19 outbreaks have been observed in homeless shelters with high proportions of infected residents and staff, including a high number of asymptomatic individuals [8, 12, 36, 37]. Homeless people should be considered particularly vulnerable to SARS-CoV-2 infection and its complications [7], and a comprehensive infection prevention strategy is required. Widespread testing, regardless of symptoms or exposure, is an important outbreak prevention measure in homeless shelters, allowing identification and isolation of persons who are asymptomatic, presymptomatic, or have only mild symptoms [17]. The frequency of screening testing can be guided by the level of community transmission. If the transmission in the community is high, facility wide (universal) testing for screening may be considered at least weekly according to CDC recommendations [17]. Universal testing in high-risk facilities will require flexible adapted approaches that have to consider also the available resources and the local recommendations. Microsimulation models may help to argue about the impact, costs, and cost-effectiveness of universal testing in homeless shelters according to various scenarios [38].

The workload for the co-researchers was high and difficult to manage together with other routine activities. Besides human resources, limited laboratory capacity for RT-PCR analysis may be a major barrier for the realization of monitoring concepts, especially if mass individual testing would be envisaged. Testing multiple samples in one approach (pooling) to screen asymptomatic people is an important strategy to consider—even if associated with challenges—when testing capacity is low and laboratory infrastructure overwhelmed [39–43]. It is also a more socially responsible strategy in regard to limited testing capacity globally [44]. The effect of testing to prevent SARS-CoV-2 transmission depends largely on the frequency of testing, as well as the turnaround time of the test result and early isolation [45, 46]. A timely test result is also relevant due to the fluctuation of shelter residents and the fact that some people may no longer be contactable later. SARS-CoV-2 antigen rapid (point-of-care) tests offer significant advantages in these respects, and they are especially useful to detect infected persons within the first week of symptom onset and those with high viral load [29]. The decreased sensitivity of antigen tests might be offset if the tests are repeated more frequently [28]. Screening concepts based on antigen rapid testing (with RT-PCR confirmatory testing) were introduced in homeless shelters and other facilities for homeless people in Berlin as from November 2020.Testing strategies should be implemented in a way that protects privacy and confidentiality. The vulnerability of the homeless population in terms of discrimination, social exclusion, residency status and resulting dependencies has to be considered in program planning. Especially in obtaining informed consent, all relevant information should be provided in an appropriate and understandable way respecting the autonomy of the shelter resident. Testing results should not be a barrier to accessing homeless services [17]. Adequate housing options are required for isolation of positive tested homeless people, addressing also possible complex needs due to chronic diseases or drug abuse.

Screening testing for COVID-19 is only one component of a required comprehensive infection prevention strategy. The German Standing Committee on Vaccination (STIKO) recommended a COVID-19 vaccine prioritization for residents and staff in community shelters, and a vaccination campaign started at homeless services sites in Berlin in March 2021 [47, 48]. SARS-CoV-2 testing priorities may shift to focus on unvaccinated homeless people and shelter staff.

## Conclusions

This study demonstrated that with appropriate efforts voluntary, regular universal testing for SARS-CoC-2 is feasible in homeless shelters. In our opinion, there are key points for successful implementation. The value of monitoring COVID-19 has to be emphasized to promote understanding and acceptability of testing, as residents may have other, more pending basic needs, especially during the winter season. Language barriers must be specially addressed, including the use of digital formats. A less invasive sampling method will result in higher compliance for regular swab testing. Self-testing with assistance, like undertaken in this study, also requires significantly less resources of qualified staff and personal protective equipment. In the current situation of a pandemic, monitoring concepts will have to accept a possible lower sensitivity of the applied methods in order to be feasible and to allow screening of individuals without symptoms on a broader scale who might spread SARS-CoV-2. Universal testing of high-risk facilities should be considered according to the level of community transmission and the available resources. Finally, participatory approaches should be sought with monitoring strategies that consider homeless people’s needs and life situation.

## Supplementary information

**Additional file 1.** Visual instruction leaflet for self-collection of saliva.

**Additional file 2.** Visual instruction leaflet for a self-collected swab of tongue, buccal mucosa and anterior nares.

**Additional file 3.** Description of the coding tree for the qualitative analysis.

**Additional file 4.** Responses to the consolidated criteria for reporting qualitative research (COREQ).

## Data Availability

The datasets generated and/or analyzed during the current study are not publicly available due containing information that could compromise the shelter residents’ privacy/consent but are available from the corresponding author on reasonable request.

## Abbreviations

COVID-19: Coronavirus disease 2019
SARS-CoV-2: Severe acute respiratory syndrome coronavirus type 2
RT-PCR: Reverse transcription-polymerase chain reaction
IQR: Interquartile range
CDC: Centers for Disease Control and Prevention

## References

[1] Aldridge RW, Story A, Hwang SW, Nordentoft M, Luchenski SA, Hartwell G (2018). Morbidity and mortality in homeless individuals, prisoners, sex workers, and individuals with substance use disorders in high-income countries: a systematic review and meta-analysis. Lancet.

[2] Baggett TP, Hwang SW, O’Connell JJ, Porneala BC, Stringfellow EJ, Orav EJ (2013). Mortality among homeless adults in Boston: shifts in causes of death over a 15-year period. JAMA Intern Med.

[3] Fazel S, Geddes JR, Kushel M (2014). The health of homeless people in high-income countries: descriptive epidemiology, health consequences, and clinical and policy recommendations. Lancet.

[4] Tsai J, Wilson M (2020). COVID-19: a potential public health problem for homeless populations. Lancet Public Health.

[5] Cumming C, Wood L, Davies A (2020). People experiencing homelessness urgently need to be recognised as a high risk group for COVID-19. Health Promot J Austr..

[6] Zhou F, Yu T, Du R, Fan G, Liu Y, Liu Z (2020). Clinical course and risk factors for mortality of adult inpatients with COVID-19 in Wuhan, China: a retrospective cohort study. Lancet.

[7] Richard L, Booth R, Rayner J, Clemens KK, Forchuk C, Shariff SZ (2021). Testing, infection and complication rates of COVID-19 among people with a recent history of homelessness in Ontario, Canada: a retrospective cohort study. CMAJ Open.

[8] Tobolowsky FG, E, Self J, et al. COVID-19 Outbreak among three affiliated homeless service sites—King County, Washington, 2020. Morbidity and Mortality Weekly Report, May 1, 2020/Vol 69/No 17. 10.15585/mmwr.mm6917e2 PMC7206987 32352954

[9] Maremmani AG, Bacciardi S, Gehring ND, Cambioli L, Schutz C, Jang K (2017). Substance Use Among Homeless Individuals With Schizophrenia and Bipolar Disorder. J Nerv Ment Dis.

[10] Wood LJ, Davies AP, Khan Z (2020). COVID-19 precautions: easier said than done when patients are homeless. Med J Aust.

[11] Roederer T, Mollo B, Vincent C, Nikolay B, Llosa AE, Nesbitt R (2021). Seroprevalence and risk factors of exposure to COVID-19 in homeless people in Paris, France: a cross-sectional study. Lancet Public Health.

[12] Mohsenpour A, Bozorgmehr K, Rohleder S, Stratil J, Costa D (2021). SARS-Cov-2 prevalence, transmission, health-related outcomes and control strategies in homeless shelters: Systematic review and meta-analysis. EClinicalMedicine.

[13] Baggett TP, Keyes H, Sporn N, Gaeta JM (2020). Prevalence of SARS-CoV-2 Infection in Residents of a Large Homeless Shelter in Boston. JAMA.

[14] Ly TDA, Nguyen NN, Hoang VT, Goumballa N, Louni M, Canard N (2021). Screening of SARS-CoV-2 among homeless people, asylum-seekers and other people living in precarious conditions in Marseille, France, March-April 2020. Int J Infect Dis.

[15] Yoon JC, Montgomery MP, Buff AM, Boyd AT, Jamison C, Hernandez A (2020). COVID-19 prevalence among people experiencing homelessness and homelessness service staff during early community transmission in Atlanta, Georgia, April-May 2020. Clin Infect Dis..

[16] Lewer D, Braithwaite I, Bullock M, Eyre MT, White PJ, Aldridge RW (2020). COVID-19 among people experiencing homelessness in England: a modelling study. Lancet Respir Med.

[17] CDC. Interim Guidance for SARS-CoV-2 Testing in Homeless Shelters and Encampments. https://www.cdc.gov/coronavirus/2019-ncov/community/homeless-shelters/testing.html. Accessed 15 Sept 2021.

[18] The National Low Income Housing Coalition. 3 May 2020. https://nlihc.org/coronavirus-and-housing-homelessness/shelter-closings. Accessed 15 Sept 2021.

[19] Conway B, Truong D, Wuerth K (2020). COVID-19 in homeless populations: unique challenges and opportunities. Future Virol.

[20] Farha L. United Nation Human Rights; 2020. COVID-19 Guidance Note, protection for those living in homelessness special rapporteur on the right to adequate housing. 2 April. https://www.ohchr.org/Documents/Issues/Housing/SR_housing_COVID-19_guidance_homeless.pdf. Accessed 15 Sept 2021.

[21] Housing and Land Rights Network (HLRN) 2020. Need for Special measures to check spread of COVID-19 among homeless and other inadequately-housed persons. https://www.hlrn.org.in/documents/Press_Release_COVID19.pdf. Accessed 15 Sept 2021.

[22] CDC. 2020. Interim Guidance for Homeless Service Providers to Plan and Respond to Coronavirus Disease 2019 (COVID-19). https://www.cdc.gov/coronavirus/2019-ncov/community/homeless-shelters/plan-prepare-respond.html. Accessed 15 Sept 2021.

[23] CDC. 2020. Interim Guidance on Unsheltered Homelessness and Coronavirus Disease 2019 (COVID-19) for Homeless Service Providers and Local Officials. https://www.cdc.gov/coronavirus/2019-ncov/community/homeless-shelters/unsheltered-homelessness.html. Accessed 15 Sept 2021.

[24] The European Federation of National Organisations Working with the Homeless (FEANTSA). COVID-19: “Staying Home” Not an Option for People Experiencing Homelessness. FEANTSA; 2020. https://www.feantsa.org/en/news/2020/03/18/covid19-staying-home-not-an-option-for-people-experiencing-homelessness?bcParent = 26.91. Accessed 15 Sept 2021.

[25] Bundesarbeitsgemeinschaft Wohnungslosenhilfe e.V. (BAG W) fordert Schutzmaßnahmen für Wohnungslose; https://www.bagw.de/de/presse/index~177.html. Accessed 20 Sept 2020.

[26] Press release of Senatsverwaltung für Integration, Arbeit und Soziales Berlin, 11 May 2020. https://www.berlin.de/sen/ias/presse/pressemitteilungen/2020/pressemitteilung.930275.php. Accessed 20 Sept 2020.

[27] Sethuraman N, Jeremiah SS, Ryo A (2020). Interpreting diagnostic tests for SARS-CoV-2. JAMA..

[28] CDC. Interim guidance for antigen testing for SARS-CoV-2. Updated Sept. 9, 2021. https://www.cdc.gov/coronavirus/2019-ncov/lab/resources/antigen-tests-guidelines.html. Accessed 15 Sept 2021.

[29] Brummer LE, Katzenschlager S, Gaeddert M, Erdmann C, Schmitz S, Bota M (2021). Accuracy of novel antigen rapid diagnostics for SARS-CoV-2: A living systematic review and meta-analysis. PLoS Med.

[30] CDC. Interim Guidelines for Collecting, Handling, and Testing Clinical Specimens for COVID-19. https://www.cdc.gov/coronavirus/2019-ncov/lab/guidelines-clinical-specimens.html. Accessed 20 Sept 2020.

[31] European Centre for Disease Prevention and Control. Diagnostic testing and screening for SARS-CoV-2. https://www.ecdc.europa.eu/en/covid-19/latest-evidence/diagnostic-testing. Accessed 20 Sept 2020.

[32] Berliner Kältehilfe. Informationen. https://kaeltehilfe-berlin.de/images/KHT_Periodenauswertung_2020_-_2021.pdf. Accessed 15 Sept 2021.

[33] Gale NK, Heath G, Cameron E, Rashid S, Redwood S (2013). Using the framework method for the analysis of qualitative data in multi-disciplinary health research. BMC Med Res Methodol.

[34] German Law on the prevention and control of infectious diseases in humans [Gesetz zur Verhütung und Bekämpfung von Infektionskrankheiten beim Menschen]. https://www.gesetze-im-internet.de/ifsg/. Accessed 15 Sept. 2021.

[35] Robert Koch Institute, COVID-19 Situation Report 13/09/2020 https://www.rki.de/DE/Content/InfAZ/N/Neuartiges_Coronavirus/Situationsberichte/Sept_2020/2020-09-13-en.pdf. Accessed 20 Sept 2020.

[36] Mosites E, Parker EM, Clarke KEN, Gaeta JM, Baggett TP, Imbert E (2020). Assessment of SARS-CoV-2 Infection Prevalence in Homeless Shelters - Four U.S. Cities, March 27-April 15, 2020. MMWR Morb Mortal Wkly Rep.

[37] Baggett TP, Keyes H, Sporn N, Gaeta JM (2020). Prevalence of SARS-CoV-2 infection in residents of a large homeless shelter in Boston. JAMA..

[38] Baggett TP, Scott JA, Le MH, Shebl FM, Panella C, Losina E (2020). Clinical Outcomes, Costs, and Cost-effectiveness of Strategies for Adults Experiencing Sheltered Homelessness During the COVID-19 Pandemic. JAMA Netw Open.

[39] Lohse S, Pfuhl T, Berko-Gottel B, Gartner B, Becker SL, Schneitler S (2020). Challenges and issues of SARS-CoV-2 pool testing–authors’ reply. Lancet Infect Dis..

[40] Lee J, Kim SY, Sung H, Lee SW, Lee H, Roh KH (2020). Challenges and issues of SARS-CoV-2 pool testing. Lancet Infect Dis..

[41] Eberhardt JN, Breuckmann NP, Eberhardt CS (2020). Challenges and issues of SARS-CoV-2 pool testing. Lancet Infect Dis..

[42] Mishra B, Behera B, Mohanty M, Ravindra A, Ranjan J (2020). Challenges and issues of SARS-CoV-2 pool testing. Lancet Infect Dis..

[43] Lohse S, Pfuhl T, Berko-Gottel B, Rissland J, Geissler T, Gartner B (2020). Pooling of samples for testing for SARS-CoV-2 in asymptomatic people. Lancet Infect Dis..

[44] Majid F, Omer SB, Khwaja AI (2020). Optimising SARS-CoV-2 pooled testing for low-resource settings. Lancet Microbe.

[45] Larremore DB, Wilder B, Lester E, Shehata S, Burke JM, Hay JA (2021). Test sensitivity is secondary to frequency and turnaround time for COVID-19 screening. Sci Adv..

[46] Mina MJ, Parker R, Larremore DB (2020). Rethinking Covid-19 Test Sensitivity - A Strategy for Containment. N Engl J Med.

[47] Vygen-Bonnet S, Koch J, Bogdan C, Harder T, Heininger U, Kling K, et al. Beschluss und Wissenschaftliche Begründung der Ständigen Impfkommission (STIKO) für die COVID-19-Impfempfehlung. Epid Bull 2021;2:3-63. 10.25646/7755.2.

[48] Vaccination start for homeless people in Berlin. New information 24 March 2021. https://www.berlin.de/aktuelles/berlin/6485620-958092-impfstart-fuer-obdachlose-.html.

