# Supplementary figures and images for: Monitoring for COVID-19 by universal testing in a homeless shelter in Germany: a prospective feasibility cohort study
Source: BMC Infect Dis. 2021 Dec 11;21:1241. doi: 10.1186/s12879-021-06945-4 (PMC8665323; doi:10.1186/s12879-021-06945-4)

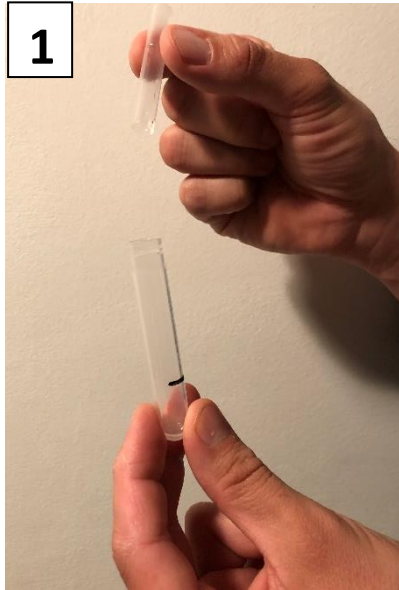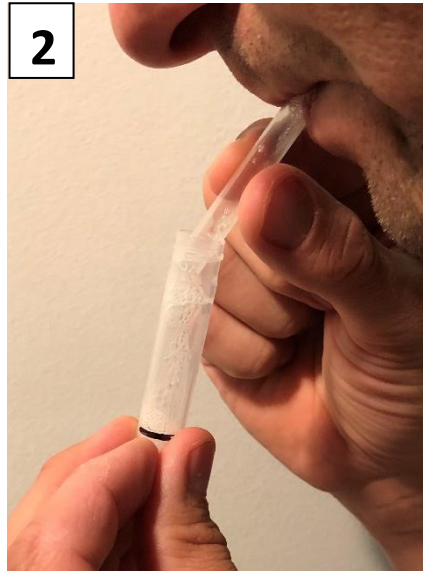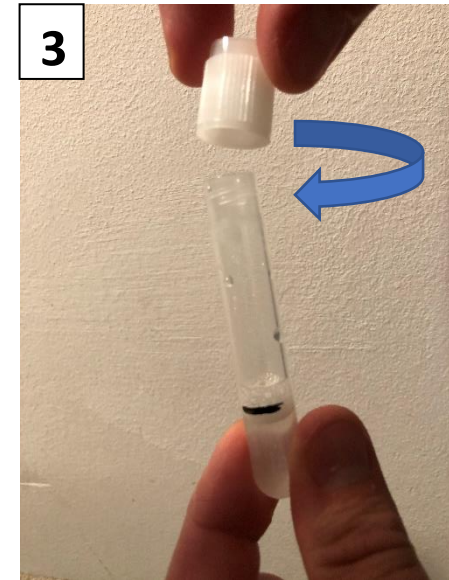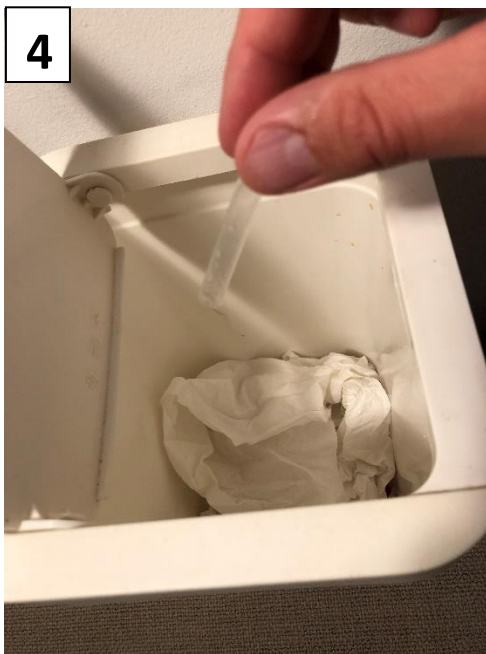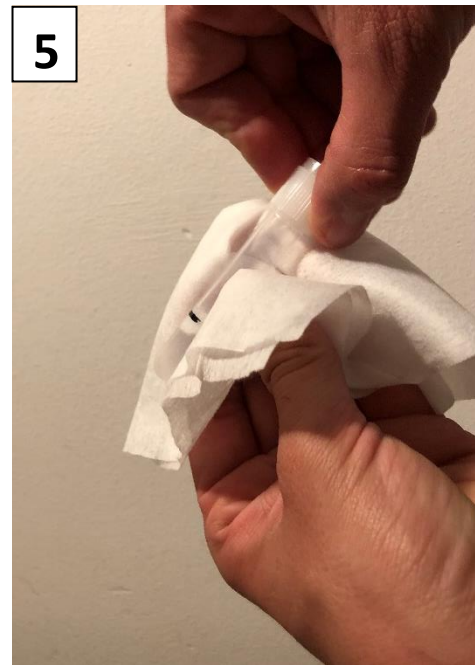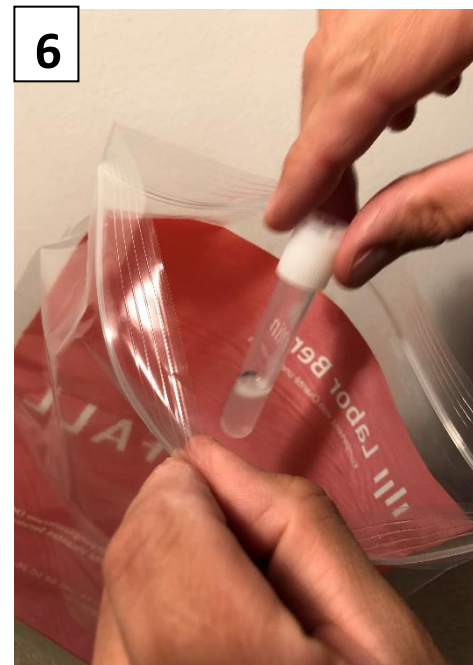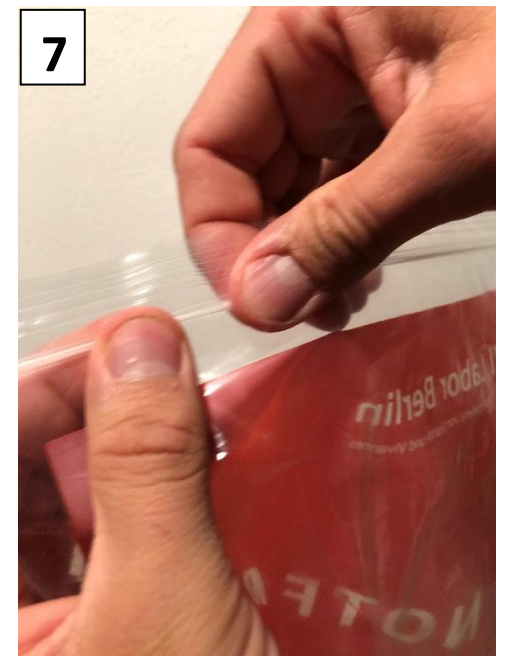

Supplement: Supplementary file 1 — Additional file 1. Visual instruction leaflet for self-collection of saliva. [file 12879_2021_6945_MOESM1_ESM.pdf]

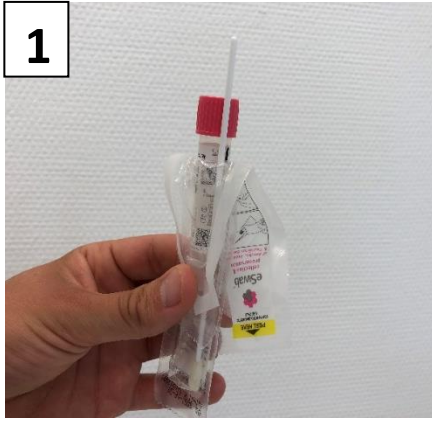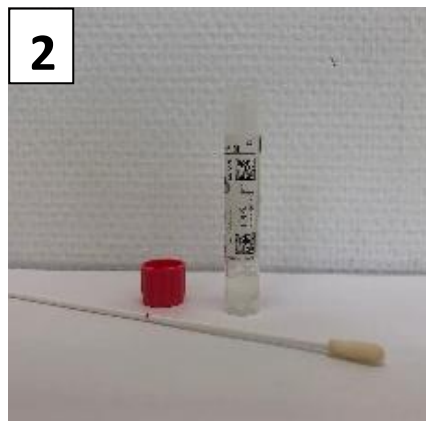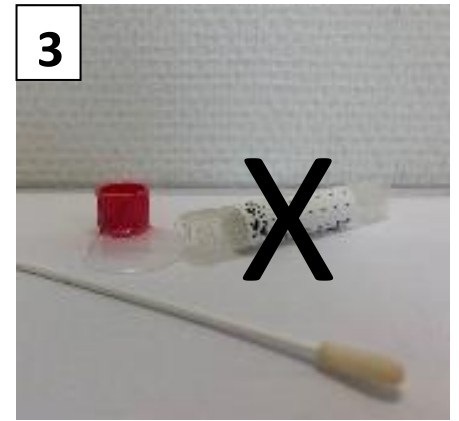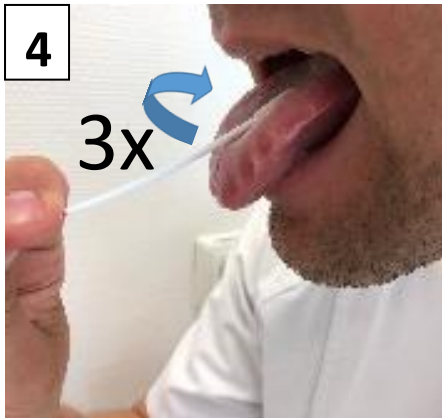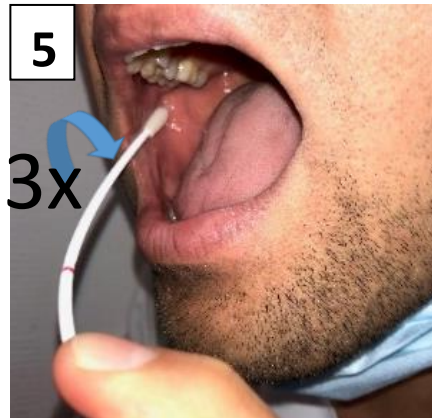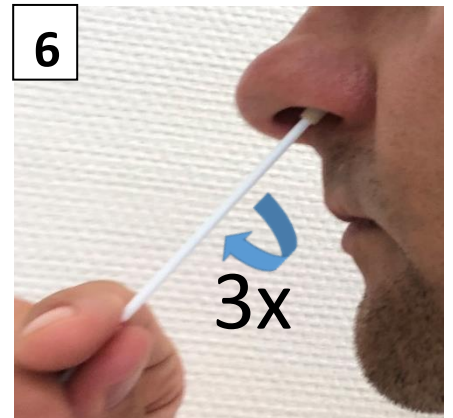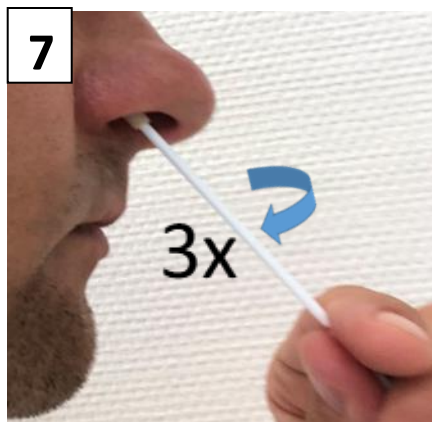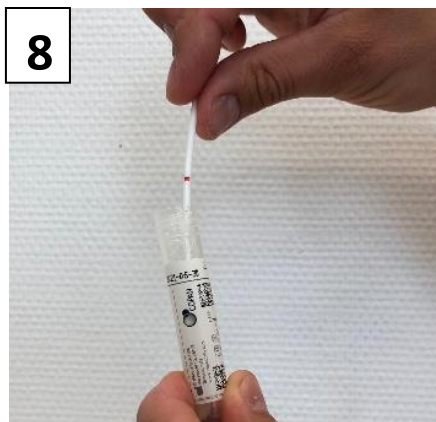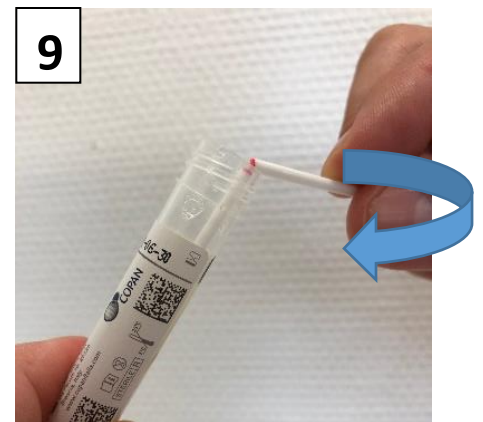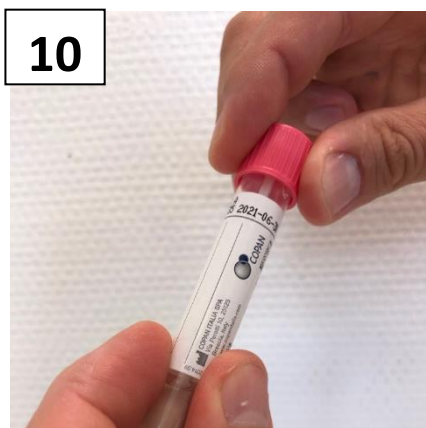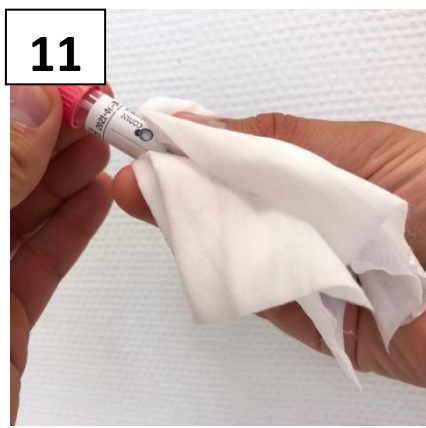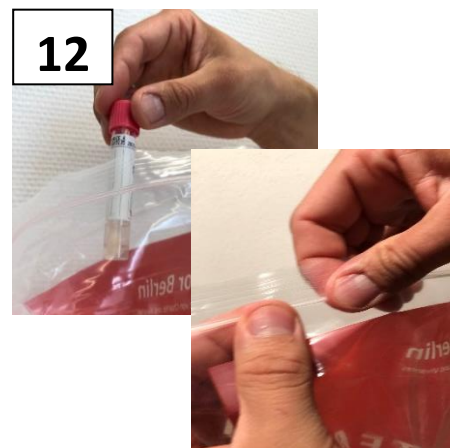

Supplement: Supplementary file 2 — Additional file 2. Visual instruction leaflet for a self-collected swab of tongue, buccal mucosa and anterior nares. [file 12879_2021_6945_MOESM2_ESM.pdf]
