# Supplementary material for: Monitoring for COVID-19 by universal testing in a homeless shelter in Germany: a prospective feasibility cohort study
Source: BMC Infect Dis. 2021 Dec 11;21:1241. doi: 10.1186/s12879-021-06945-4 (PMC8665323; doi:10.1186/s12879-021-06945-4)
Supplement: Supplementary file 3 — Additional file 3. Description of the coding tree for the qualitative analysis. [file 12879_2021_6945_MOESM3_ESM.pdf]

Additional file 3: Description of the coding tree for the qualitative analysis

Category	Deductive code	Inductive code	Description
Implementation		Preparation	Preparation in terms of clear work agreements, responsibilities and procedures
		Communication	Transparent communication within the team
	Team composition		Number, qualifications, and language skills of the project team
		Trust	Include persons in the team that already established trust with the participants
		Time	Time needed for training, meetings, coordination, and preparation for sample collection
	Informed consent		Time, language, and attitude of approach
	Sample collection		Time, language, and attitude of approach
Barriers for participation	Languages		Languages spoken and understood by the participants and the project team
	Priorities		Life situation of the participants may cause other priorities for them other than participation in a monitoring
	Personal data		Personal data will be given to the local health authority in case of a positive test result. That might affect unstable residence status of participants.
		Signature	Giving a signature can be a barrier for people in vulnerable situations
		Timing	Time for obtaining informed consent that provides privacy and does not interfere with other activities
		Written test result	Provision of written test result would be a benefit.
Sample collection		Handling in general	Prepared lists and material for easy procedure at a convenient time, plan enough time for each sample collection
	Handling of salvia		Including aspects such as time, hygiene, preparation, and comfort
	Handling of swab		Including aspects such as time, hygiene, preparation, and comfort

		Assistance	Assistance of sample collection rather than use of instruction leaflet, patience and understanding for multiple assistance
Informed consent	Content of information		Information about all aspects of the study and consequences of a positive test result in a way and language that participants understand
	Approach		Respect and understanding as well as knowledge of the life situation of the participants
	Inquiries by participants		Questions and priorities of the participants
	Benefit (participant)		Individuals without health insurance have no easy access to testing, safety for the community
	Benefit (shelter)		Safety in the shelter at times of testing
		Mode	Alternative modes such as video, and audio formats
Recommendations for further implementation in over-night shelters during winter		Valuation of the participants	Participant's live situation and circumstances
		Acknowledge language diversity	Multiple languages are being spoken by participants
		Barrier: Time of monitoring	Consideration of seasonally dependent workload
		Barrier: Substance use	Substance and alcohol use can be a barrier for participation
		Barrier: Setting	Workload in over-night shelters during winter is different and higher compared to 24/7 shelters, staff fluctuates a lot, smaller premises
		Barrier: Priorities	During winter, priorities shift to basic needs
	COVID-19 knowledge		Knowledge about covid-19 and need for more information
	Adapted material		Adaption of documents for participants to literacy and visual ability of participants
		Communication of test results	Communication of test results vie telephone or central contact point
