# Supplementary material for: Monitoring for COVID-19 by universal testing in a homeless shelter in Germany: a prospective feasibility cohort study
Source: BMC Infect Dis. 2021 Dec 11;21:1241. doi: 10.1186/s12879-021-06945-4 (PMC8665323; doi:10.1186/s12879-021-06945-4)
Supplement: Supplementary file 4 — Additional file 4. Responses to the consolidated criteria for reporting qualitative research (COREQ). [file 12879_2021_6945_MOESM4_ESM.pdf]

Additional file 4: Response to the consolidated criteria for reporting qualitative studies (COREQ): 32-item checklist[1]

No	Item	Guide questions/ description	Response
**Domain 1: Research team and reflexivity**			
Personal Characteristics			
1.	Interviewer/ facilitator	Which author/s conducted the interview or focus group?	Lindner AK, Sarma N
2.	Credentials	What were the researcher's credentials? *E.g. PhD, MD*	**Principle researchers**<br>Lindner AK, MD, MSc (TMIH), DTM&H<br><br>Sarma N, M.A. South Asian History, Masters of Public Health
3.	Occupation	What was their occupation at the time of the study?	**Principle researchers**<br>Lindner AK, clinician and researcher at Charité-Universitätsmedizin, Institute of Tropical Medicine and International Health, Berlin, Germany<br><br>Sarma N, associate researcher at Department of Infectious Disease Epidemiology, Robert Koch Institute, Berlin, Germany<br><br>**Co-Researchers involved in the qualitative analysis**<br>Svetlana Krasovski-Nikiforovs, Coordinator of all medical projects at Berliner Stadtmission<br><br>*Staff of the 24/7 shelter*<br>Theresa Hellmund, Coordinator<br>Anna Behnke, Coordinator<br>Wojciech Greh

No	Item	Guide questions/ description	Response
			Leon Hoffmann<br>Dominika Jurasik<br>Miriam Luchterhand<br>Jonas Kalmbach<br>Valeska Steinert<br>Sophie Rothe<br>Uldis Stukmanis<br>Franek Machowski<br>Wojciech Greh<br>Olga Nikolai<br><br>*Staff of quarantine station*<br>Luise Marie Rust, Coordinator<br>Sarah M. Klaes<br>Merle Hoerig<br>Sophia Monert<br>Derrick Akechu Wouba<br>Gabriela Aldama<br>Oskar Herbst<br>Pearl von Herder<br>Sophie Hilt<br>Gabriela Aldama<br><br>All members of the research team were native or bilingual German speakers.
4.	Gender	Was the researcher male or female?	Seventeen researchers were female and nine researchers male.
5.	Experience and training	What experience or training did the researcher have?	**Principle researchers**<br>Lindner, A: specialist in Infectious Diseases and Tropical Medicine. Master in Tropical Medicine and International Health, and Diploma of Tropical Medicine and Hygiene at the London School of Hygiene and Tropical Medicine. Investigator in several clinical studies during the last 8 years, principle investigator of another ongoing

No	Item	Guide questions/ description	Response
			clinical study. Certification in Good Clinical Practice (GCP).<br><br>Sarma, N: 6 years of experience leading qualitative research, and collecting and analyzing qualitative data in public health research, as an associate researcher and project coordinator; 3 years of experience coordinating participatory health research projects; Certification course Participatory Social Research, Catholic University of Applied Sciences/International Collaboration for Participatory Health Research (ICPHR); Certification course Good Health Research Practice, Institute of Public Health, Bangalore, India, 10 years of experience volunteering in the medical projects of Berliner Stadtmission as a nurse.
Relationship with participants			
6.	Relationship established	Was a relationship established prior to study commencement?	**Process evaluation**<br>Sarma N had already established relationships with single members of the co-research team due to her voluntary work in medical projects of Berliner Stadtmission.<br><br>**Monitoring**<br>Sarma N and Krasovski-Nikiforovs S might have established relationships with residents in single cases, who were patients of medical projects of Berliner Stadtmission.<br><br>Co-researchers of the 24/7 shelter had established a relationship with most of the participants/residents through their daily interaction.

No	Item	Guide questions/ description	Response
			Staff of the quarantine unit did not necessarily have an established relationship with the residents. Residents were approached usually by mixed teams of the 24/7 shelter and the quarantine unit.
7.	Participant knowledge of the interviewer	What did the participants know about the researcher? e.*g. personal goals, reasons for doing the research*	**Process evaluation**<br>An introduction of all persons took place at the beginning of each meeting. The co-researches knew the principle researchers in person from the beginning.<br><br>**Monitoring**<br>Through the standardized oral information and from the written forms, shelter residents knew that the study was a cooperation between Berliner Stadtmission and Charité University Hospital Berlin. The Charité is known to many homeless people for the medical care. The shelter residents knew the co-research team in person due to the recruitment procedure and sample collection.
8.	Interviewer characteristics	What characteristics were reported about the interviewer/facilitator? e.g. *Bias, assumptions, reasons and interests in the research topic*	**Process evaluation**<br>We tried to create a positive and welcoming atmosphere during project team meetings and the focus group. We encouraged co-researchers to freely and critically express their ideas and perceptions about their experiences of the study with regard to future implementation.<br><br>**Monitoring**<br>The co-researchers reported to the shelter residents that we were a multidisciplinary team, interested in identifying barriers and

No	Item	Guide questions/ description	Response
			facilitators of COVID-19 monitoring, with the goal of improving COVID-19 prevention.
**Domain 2: study design**			
Theoretical framework			
9.	Methodological orientation and Theory	What methodological orientation was stated to underpin the study? *e.g. grounded theory, discourse analysis, ethnography, phenomenology, content analysis*	**Process evaluation**<br>Our process evaluation used mixed methods and was based on the framework method. We cited Gale et al. (ref. 29). To integrate knowledge from the shelter staff, we used a participatory approach for the development of the study concept and the evaluation sheets. Our methodology had both inductive elements (developing new codes based on qualitative data) and deductive exploration of predefined themes (feasibility). Final results were discussed and put into context in the final focus group.
Participant selection			
10.	Sampling	How were participants selected? *e.g. purposive, convenience, consecutive, snowball*	**Process evaluation**<br>All co-researchers were part of the process evaluation. The co-research team chose two members who were in charge of the data entry.<br><br>**Monitoring**<br>All residents of the shelter were considered as potential participants.
11.	Method of approach	How were participants approached? e*.g. face-to-face, telephone, mail, email*	**Process evaluation**<br>The principle researchers approached the co-researchers mainly by phone, video call or email due to contact restrictions during the COVID-19 pandemic. Documents were digitally developed and shared. Only

No	Item	Guide questions/ description	Response
			accompanying daily meetings during the first week, trainings and the final focus group were face-to-face-meetings.<br><br>**Monitoring**<br>Residents were approached face-to-face by the co-researchers, if possible, in their native languages.
12.	Sample size	How many participants were in the study?	**Process evaluation**<br>The planning of the study and the accompanying daily meetings during the first week of implementation took place in small groups of 4-8 participants including the coordinators and principle researchers.<br><br>Focus group participants: 18 co-researchers<br>Participants of final evaluation questionnaire: 12 co-researchers<br>Total: 22 co-researchers<br><br>**Monitoring**<br>Total: 51 shelter residents
13.	Non-participation	How many people refused to participate or dropped out? Reasons?	**Process evaluation**<br>A total of 4 co-researchers did not participate in the process evaluation probably because it was not during their shift.<br>The final evaluation sheet was given to all co-researchers, but 10 co-researchers did not return it.<br><br>**Monitoring**<br>The co-researchers kept records of refusals. The reasons of the homeless people for refusing participation are shown in Fig. 1.

No	Item	Guide questions/ description	Response
Setting			
14.	Setting of data collection	Where was the data collected? e*.g. home, clinic, workplace*	**Process evaluation**<br>Data was collected during meetings in the garden of the shelter.<br><br>**Monitoring**<br>Data was collected in the eating area, garden and sleeping rooms of the shelter as well as in areas around the shelter where residents spent their day.
15.	Presence of non-participants	Was anyone else present besides the participants and researchers?	No.
16.	Description of sample	What are the important characteristics of the sample? *e.g. demographic data, date*	**Process evaluation**<br>All participants were co-researchers.<br><br>**Monitoring**<br>The baseline characteristics of residents approached for participation are shown in Table 2.
Data collection			
17.	Interview guide	Were questions, prompts, guides provided by the authors? Was it pilot tested?	The evaluation sheets were further developed and modified during implementation. The focus group guide and final evaluation sheet were developed by the principle researchers. They were not piloted.
18.	Repeat interviews	Were repeat interviews carried out? If yes, how many?	No repeat interviews were carried out.

No	Item	Guide questions/ description	Response
19.	Audio/visual recording	Did the research use audio or visual recording to collect the data?	No.
20.	Field notes	Were field notes made during and/or after the interview or focus group?	Field notes were made both during and after the meetings and focus group.
21.	Duration	What was the duration of the interviews or focus group?	The duration of the meetings and focus group was 1.5 – 2 hours each.
22.	Data saturation	Was data saturation discussed?	Data saturation was not explicitly discussed.
23.	Transcripts returned	Were transcripts returned to participants for comment and/or correction?	Transcripts were not returned for comments/correction.
**Domain 3: analysis and findings**			
Data analysis			
24.	Number of data coders	How many data coders coded the data?	2 coders
25.	Description of the coding tree	Did authors provide a description of the coding tree?	The coding tree is provided as Additional file 3.
26.	Derivation of themes	Were themes identified in advance or derived from the data?	Themes were mainly identified in advance and to a small extend derived from the data.
27.	Software	What software, if applicable, was used to manage the data?	No software was used.

No	Item	Guide questions/ description	Response
28.	Participant checking	Did participants provide feedback on the findings?	**Process evaluation**<br>Co-researchers provided feedback on the findings of the study and the process evaluation during the final focus group.<br><br>**Monitoring**<br>The findings were not discussed with the shelter residents.
Reporting			
29.	Quotations presented	Were participant quotations presented to illustrate the themes / findings? Was each quotation identified? e.*g. participant number*	**Process evaluation**<br>We presented several quotations of the co-researchers to illustrate the themes. Each quotation was identified from an individual participant (participants numbers not shown).
30.	Data and findings consistent	Was there consistency between the data presented and the findings?	There was high consistency between the data presented and the findings.
31.	Clarity of major themes	Were major themes clearly presented in the findings?	Major themes were clearly presented in the findings.
32.	Clarity of minor themes	Is there a description of diverse cases or discussion of minor themes?	We have included a description of diverse cases, as well as of minor themes.

[1] Tong A, Sainsbury P, Craig J. Consolidated criteria for reporting qualitative research (COREQ): a 32-item checklist for interviews and focus groups, *International Journal for Quality in Health Care*, Volume 19, Issue 6, December 2007, Pages 349–357, https://doi.org/10.1093/intqhc/mzm042
